# Beetles, ants, wasps, or flies? An ethnobiological study of edible insects among the *Awajún* Amerindians in Amazonas, Peru

**DOI:** 10.1186/s13002-018-0252-5

**Published:** 2018-08-09

**Authors:** Rubén Casas Reátegui, Lukas Pawera, Pablo Pedro Villegas Panduro, Zbynek Polesny

**Affiliations:** 1 Department of Crop Sciences and Agroforestry, Faculty of Tropical AgriSciences, Czech University of Life Sciences Prague, Kamýcká 129, 165 00 Praha 6 - Suchdol, Czech Republic; 2 Departamento Agroforestal Acuícola, Facultad de Ingeniería y Ciencias Ambientales, Universidad Nacional Intercultural de la Amazonia, Carretera a San Jose K.m. 0.5, Yarinacocha, Pucallpa, Peru; 3 Departamento de Ciencas Agricolas, Facultad de Ciencias Agropecuarias, Universidad Nacional de Ucayali, Carretera Federico Basadre K.m. 6.00, Pucallpa, Peru

**Keywords:** Entomophagy, Ethnoentomology, Food, Insect, Peruvian Amazon, Traditional knowledge

## Abstract

**Background:**

Insects are known to be able to provide valuable nutrients to indigenous populations across the Amazon. However, studies on traditional insect use in the Peruvian Amazon are scarce. This study documents edible insect diversity and characterizes their food and collection patterns in eight *Awajún* communities in the Peruvian Amazon. Additionally, we reviewed what has been known to date about the nutrient composition of the documented species.

**Methods:**

The survey was conducted among the *Awajún* populations living in the Huampami, Paisa, Achu, and Tseasim communities in the Cenepa district and the Shijap, San Mateo, Kusu, and Listra communities in the Imaza district. Data collection was conducted through a freelisting exercise complemented by a semi-structured inquiry form in the *Awajún* language. In total, 104 informants (72 men and 32 women) aged between 16 to 73 years were interviewed.

**Results:**

The *Awajún* people use at least 12 insect species, with *Rhynchophorus palmarum*, *Atta cephalotes*, and *Rhinostomus barbirostris* being the most important ones. Beetles of the family Curculionidae represent the culturally most salient taxon. In the more accessible and developed Imaza district, the *Awajún* tend to eat almost exclusively *R. palmarum*, while in the more isolated and preserved Cenepa district, the community’s preferences are linked with more species. Although men are the main insect collectors, women cited more edible insects on average. The insects are eaten mainly roasted or raw. Further use patterns and differences between the districts are discussed.

**Conclusion:**

Traditional knowledge related to edible insects and the ecosystems they occur in is widespread among the *Awajún* populations, and insects still represent an important part of the indigenous food system. This ethnobiological survey discovered five species that are newly recorded as edible insects. Chemical composition of insects deemed edible by the *Awajún* ought to be analyzed in the future and awareness about their nutritional importance should be raised to harness the potential of this underutilized yet nutrient-rich traditional food.

## Background

According to recent reports on the state of food insecurity in the world, some 795 million people globally are notoriously undernourished, with the majority living in developing countries. In Latin America and the Caribbean, undernourishment affects 34.3 million people (5.5% of the population) [1]. The nutritional profile in Peru remains alarming. In 2016, 13.1% of Peruvian children under 5 years old were undernourished, and in rural areas 41.4% suffered from anemia [2]. Edible insects can contribute as a sustainable source of high-quality protein, lipids, carbohydrates, minerals, and certain vitamins, especially B vitamins, with the exception of B12. The study of insects as food, as well as the promotion of the management of this resource to alleviate global food shortage, goes back to 1975 [3] and has become one of the main objectives of ethnoentomology [4, 5]. According to the most recent list of edible insects worldwide, 2111 insects are used as a food [6]. Previous studies of indigenous societies of the Amazon Basin showed consumption of the orders Hymenoptera, Coleoptera, and Orthoptera mainly among the indigenous groups in Brazil [7] and Colombia [8].

In Peru, the *Awajún* indigenous people live in the hills and on the river banks of Marañón, Cenepa and others. Their traditional culture was largely affected in the middle of the twentieth century when Jesuit and Protestant missionaries with governmental assistance brought education and Christianity to the area [9]. *Awajún* beliefs about the forest include that the jungle was populated with spirits and that animals or plants possess “a soul” [10]. This spiritual connection with nature was considered animism and suppressed by the evangelists [11]. Traditionally, the *Awajún* are a semi-nomadic ethnic group with activities consisting of fishing, hunting, gathering, and slash-and-burn farming. This livelihood strategy explains the complexity of the socio-ecological system, based on regular migration, which prevents depletion of hunting zones, fishing spots and land used for agriculture [12]. The *Awajún’s* primary source of dietary energy is cassava (*Manihot esculenta* Crantz) complemented with edible resources obtained through fishing, hunting, farming, and gathering [13]. These traditional indigenous community practices not only represent sustainable ways to harvest economically important resources, but they are also inevitably linked to the cultural identities [14]. Nonetheless, hunting and fishing could likely become difficult with increasingly disturbed forest areas. For example, the indigenous territories of the Peruvian Amazon have lost more than 9000 km^2^ of forest due to deforestation in 2013 [15]. A typical adult *Awajún* consumes 93% of his or her dietary energy from local food resources, and the *Awajún* traditional food system (farming, hunting, fishing, and collecting) comprises approximately 223 edible animal and plant species or varieties including three insect species representing 1% of the total food consumed [16]. Extreme poverty, social exclusion, chronic undernutrition, and anemia are the main problems affecting the *Awajún* people in Peru’s Amazonas Region. An earlier nutritional study performed in four Amazonas districts determined that 33.4% of children suffered from chronic malnutrition, while 50.2% of women of childbearing age suffered from anemia. The study deduced that these results were probably caused by an unbalanced diet based on monotonous consumption of staple foods such as cassava and bananas (plantains) and low consumption of animal proteins [17]. Although evidence exists of insect consumption as a relevant protein source for indigenous populations across the Amazon, ethnozoological studies from the Peruvian Amazon are scant. Among those, Delgado et al. [18] and Vargas et al. [19] conducted studies on the management and nutritional value of the *Rhynchophorus palmarum* L. larvae consumed by Amazonian populations such as the *Kukama Kukamiria* of the Loreto Region. Using an ethnobiological perspective [20], further studies on local insect use and management are needed, particularly when nutritional characteristics and future economic interest in these resources are considered. Moreover, due to availability, abundance, and easy reproduction, insects might be seen as an option for reducing pressure on some locally collected plants or hunted animals.

Considering the lack of studies from the Peruvian Amazon, the present ethnobiological study aimed to (1) document the diversity of edible insects consumed within the *Awajún* communities in the Amazonas Region; (2) determine the cultural importance of particular species, families and orders; (3) analyze the variety in patterns of use according to demographic factors; and (4) compare the knowledge and uses between two districts with different socio-ecological conditions. In addition, the study provides summarized information on the nutritive value of documented insects based on a survey of the available literature and food composition tables.

## Methods

### Study area

**Fig. 1:**
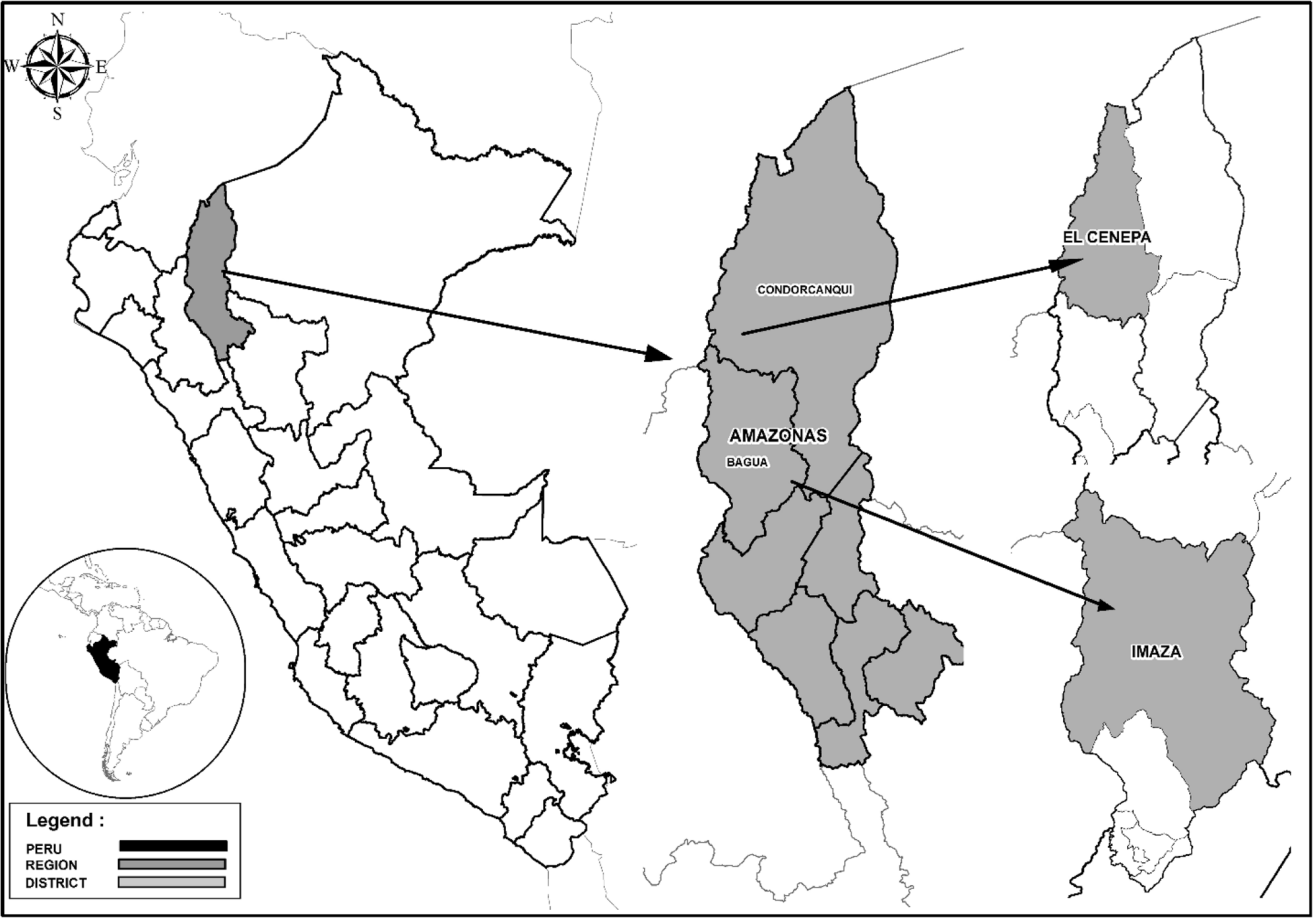
Study area map

This study was performed in eight *Awajún* communities along the upper Marañón and Cenepa rivers in the Amazonas Region in the northern Peruvian Amazon (Fig. 1). It was estimated that 43,896 *Awajún* people live in Peru, and of them, 15,767 live in Imaza and 7303 live in the Cenepa district. This study involved the Huampami, Paisa, Achu, and Tseasim communities in the Cenepa district and the Shijap, San Mateo, Kusu, and Listra communities in the Imaza district. All the communities are located in the eastern foothills of the Andes at an elevation range of 200–500 m.a.s.l., with mountains up to 1000 m.a.s.l. in close proximity. The predominant natural vegetation corresponds to the tropical wet forest and premontane tropical rainforest according to the Holdridge classification [21]. The presence of the Tuntanain and Condor ranges in part of its territory allows the population greater access to forest resources.

Demographically, the Cenepa District is populated by the *Awajún* people exclusively, whereas the Imaza District has a large mestizo population who have entered through the road to the city of Bagua Chica and who have possibly contributed to a loss of traditional *Awajún* knowledge in this district [22]. Linguistically, the *Awajún* is one of the four large ethnic/linguistic groups of the *Jivaroan* language family including *Achual*, *Awajún*, *Huambisa*, and *Jíbaro-Shuar*, located in the Marañón River basin. The participating communities have a subsistence-based economy, including swidden horticulture, supplemented extensively by livestock raising, wild-plant gathering, fishing, and bird and game hunting [9].

### Data collection and analysis

Data were gathered in the study area from June to October 2015 using field surveys. Eight villages (one village per community) were visited, and 104 people were interviewed (72 men and 32 women). The respondents’ ages ranged from 16 to 73 years with a mean age of 42.6 ± 13.3 years (median = 40.5). All respondents stated they were evangelical Christians belonging to the Nazarene church. Prior to the beginning of the research, each respondent was informed about the survey’s purpose and participated on a volunteer basis with verbal consent. Data collection was based on freelists and semi-structured questionnaires with interviews performed in the *Awajún* language [23, 24]. We asked the informants to list local insects they gather, their vernacular names, the developmental stage consumed, the mode of preparation, the gathering method, and the seasonal availability.

Whenever possible, we made entomological collections to verify the taxonomic identity of the insects mentioned. The nomenclature used follows the International Commission on Zoological Nomenclature [25]. Insect specimens were deposited in the Laboratory of Entomology of the *Universidad Nacional de Ucayali* in Pucallpa, Peru.

To determine the cultural importance of each insect, the freelists were analyzed using the Smith’s Salience Index (S) [26]. This index of a cultural domain analysis considers both a citation’s frequency and rank [27]. First, to calculate species salience values per list, each listed insect was ranked according to its order in the list (starting from 1 for the insect listed first). The ranks were then converted and divided by the total number of insects cited in the list. Composite salience was obtained by dividing the summed salience values for each insect by the number of informants (*n* = 104). The obtained Salience Index (S) was also used to calculate the overall cultural importance of insect developmental stages and taxonomic units (total salience). The relationship between the number of listed insects (dependent variable) was correlated with the independent variables (age, income, number of children) using non-parametric Spearmen correlation as the data were not normally distributed. The analysis was performed using IBM SPSS Statistics 24.

A review of insects’ nutritive values (energy value and macronutrients’ content) was derived from international and Spanish-written research articles complemented with data in available national and international food composition tables. The nutrient’s content on dry-weight basis were converted to fresh-weight basis if the value of water content was provided [28].

## Results

### Edible insects’ diversity in the *Awajún* food system

Twelve insect species belonging to three orders (Coleoptera, Diptera, Hymenoptera) and 6 families (Curculionidae, Elateridae, Formicidae, Vespidae, Scarabaeidae, and Stratiomyidae) are eaten by the *Awajún* population in the study area (Table 1). Considering biodiversity at the family level, Coleoptera were represented by three families (Curculionidae, Elateridae, and Scarabaeidae), Hymenoptera by two families (Formicidae and Vespidae), and Diptera by one family (Stratiomyidae). At the species level, the 12 edible insect species identified in our study were 6 beetles (Coleoptera), 2 wasps, 3 ants (Hymenoptera), and 1 fly (Diptera). Based on our literature review of edible insects worldwide, the present research identified 5 insect species, namely *Agelaia pallipes* Olivier, *Cephalotes atratus* Linnaeus, *Crematogaster sordidula* Nylander, *Cyphomyia auriflamma* Wiedemann, and *Strategus jugurtha* Burmeister, as new records for insects used as a food.

**Table 1:** Insect taxa and their use as food by the *Awajún* people in descending order of the Salience Index (S)

Species	Order	Family	Vernacular name	Consumption stage	Form of preparation	Gathering method	Seasonal availability	*S*	% of respondents
Rhynchophorus palmarum*	Coleoptera	Curculionidae (beetles)	*Bukín* [bu.ˈkĩn]	Larva	Grilling, roasting on stick, frying, boiling	Collecting from split palm trunk of *Mauritia flexuosa*, *Attalea phalerata*, *Astrocaryum chambira, Oenocarpus bataua* and *Bactris gasipaes*	Jan–Dec	0.83	99
*Atta cephalotes*	Hymenoptera	Formicidae (ants)	*Week* [we.ˈɛk]	Adult	Toasting, frying	Collecting ants from burned nest	Sep–Oct	0.50	88
*Rhinostomus barbirostris*	Coleoptera	Curculionidae (beetles)	Datush [ˈda.tus]	Larva	Grilling, roasting on stick	Collecting from split palm trunk of *Mauritia flexuosa*, *Oenocarpus bataua*, *Attalea phalerata* and *Bactris gasipaes*	Jan–Dec	0.47	81
*Agelaia pallipes*	Hymenoptera	Vespidae (wasps)	*Usuk ete* [u. ˈsuk ˈɛ.te]	Pupae	Roasting, toasting	Collecting eggs and nymphs from burned nest	Jun–Aug	0.37	63
*Crematogaster sordidula*	Hymenoptera	Formicidae (ants)	*Máya* [ˈma.ʝa]	Adult	Roasting	Collecting from split tree trunk of *Ochroma pyramidale*	Jan–Dec	0.35	67
*Metamasius hemipterus*	Coleoptera	Curculionidae (beetles)	Daish [ˈdai̯s]	Larva	Consumed directly (raw)	Collecting from split palm trunk of *Mauritia flexuosa*, *Attalea phalerata*, *Astrocaryum chambira* and *Bactris gasipaes*	Jan–Dec	0.35	58
Rhynchophorus palmarum*	Coleoptera	Curculionidae (beetles)	*Tsampun* [ˈt.sãm.pũn]	Adult	Toasting, grilling	Collecting from split palm trunk of *Mauritia flexuosa*, *Attalea phalerata* and *Astrocaryum chambira* and *Bactris gasipaes*	Jun–Sep	0.19	40
*Cyphomyia auriflamma*	Diptera	Stratiomyidae (flies)	*Kawat* [ka.ˈwat]	Larva	Consumed directly (raw)	Collecting from split palm trunk of *Mauritia flexuosa*, *Attalea phalerata* and *Astrocaryum* spp.	Jan–Dec	0.10	26
*Strategus jugurtha*	Coleoptera	Scarabaeidae (beetles)	*Kuru* [ˈku.ɾu]	Adult	Toasting, Grilling	Collecting from cut youth branches of *Gynerium sagittatum*	Jun–Sep	0.05	25
*Megaceras crassum*	Coleoptera	Scarabaeidae (beetles)	*Amuntai* [a.mũn̪.ˈtai̯]	Adult	Toasting, grilling	Collecting from cut youth branches of *Gynerium sagittatum*	Jun–Sep	0.03	9
UID**	Coleoptera	Elateridae (beetles)	*Chuu* [ˈʧu.u]	Larva	Consumed directly (raw)	Collecting from split palm trunk of *Mauritia flexuosa*	Jan–Dec	0.03	6
*Cephalotes atratus*	Hymenoptera	Formicidae (ants)	*Dakerae* [da.kɛ.ˈɾa.e]	Adult	Roasting	Collecting from split trunk of various host plants	Jan–Dec	0.01	4
*Mischocyttarus* spp.	Hymenoptera	Vespidae (wasps)	*Shanu* [ˈsa.nu]	Pupae	Roasting, toasting	Collecting eggs and nymphs from burned nest	Jun-Aug	0.01	1

*Both developmental stages (larvae and adults) of *Rhynchophorus palmarum* were consumed. Therefore, the species is mentioned twice in the table and the data was presented separately for each developmental stage

**UID unidentified species. The species was taxonomically identified down to family level only

### Insects’ cultural importance and socio-cultural factors

In total, 579 reports (respondent *r*, mentioned use of a species *s*) were obtained. If all communities were considered together (Table 1), the most culturally salient insects were *R. palmarum* larvae (S = 0.83), followed by *Atta cephalotes* L. adults (S = 0.50) and *Rhinostomus barbirostris* F. larvae (S = 0.47). The adult stage of *R. palmarum* was consumed although it was culturally low salient (0.19), represented the sole species of beetles consumed in different developmental stages. According to taxonomical group salience (Table 2), Coleoptera is the most culturally important order, reaching the highest average (0.28) and total Salience Index (1.95), followed by Hymenoptera and Diptera. Curculionidae is the most culturally salient family, while the Elateridae are the least important. Looking at the cultural significance of the insects’ developmental stages, larvae obtained the highest average (0.36) and total Salience Index (1.78) followed by adult and pupal stages.

**Table 2:** Cultural significance of insect taxa and developmental stages

Insect groups	No. of species	Mean S*	Total S	% of citations	% of respondents
Consumption stage					
Larva	5	0.36	1.78	48	100
Adult	5	0.19	1.13	41	96
Pupae	2	0.19	0.38	11	64
Order					
Coleoptera	6	0.28	1.95	56	100
Hymenoptera	4	0.25	1.24	39	96
Diptera	1	0.10	0.10	5	26
Family					
Curculionidae (beetles)	3	0.46	1.84	49	100
Formicidae (ants)	2	0.29	0.86	28	94
Vespidae (wasps)	2	0.19	0.38	11	63
Stratiomyidae (flies)	1	0.10	0.10	5	26
Scarabaeidae (beetles)	2	0.04	0.08	6	25
Elateridae (beetles)	1	0.03	0.03	1	6

*S Salience Index

No relationship existed between the number of listed insects and age of respondents (*r* = − 0.0803, *n* = 104, *P* > 0.05). A weak positive relationship was found between the number of reported insects and the number of children in the households (*r* = 0.223, *n* = 104, *P* < 0.05) and the monthly respondents’ income (*r* = 0.280, *n* = 104, *P* < 0.01). The proportion of insect sellers was equal in both districts (40%).

In Cenepa, women cited 4.47 ± 1.13 insect species, while men offered 4.14 ± 0.92, and in Imaza, women cited 6.82 ± 1.13 species compared to 6.3 ± 1.67 species reported by the men. However, all men together indicated 13 species, whereas women identified only 11 species.

### Comparison of insect diversity, knowledge, and consumption in the Cenepa and Imaza Districts

**Fig. 2:**
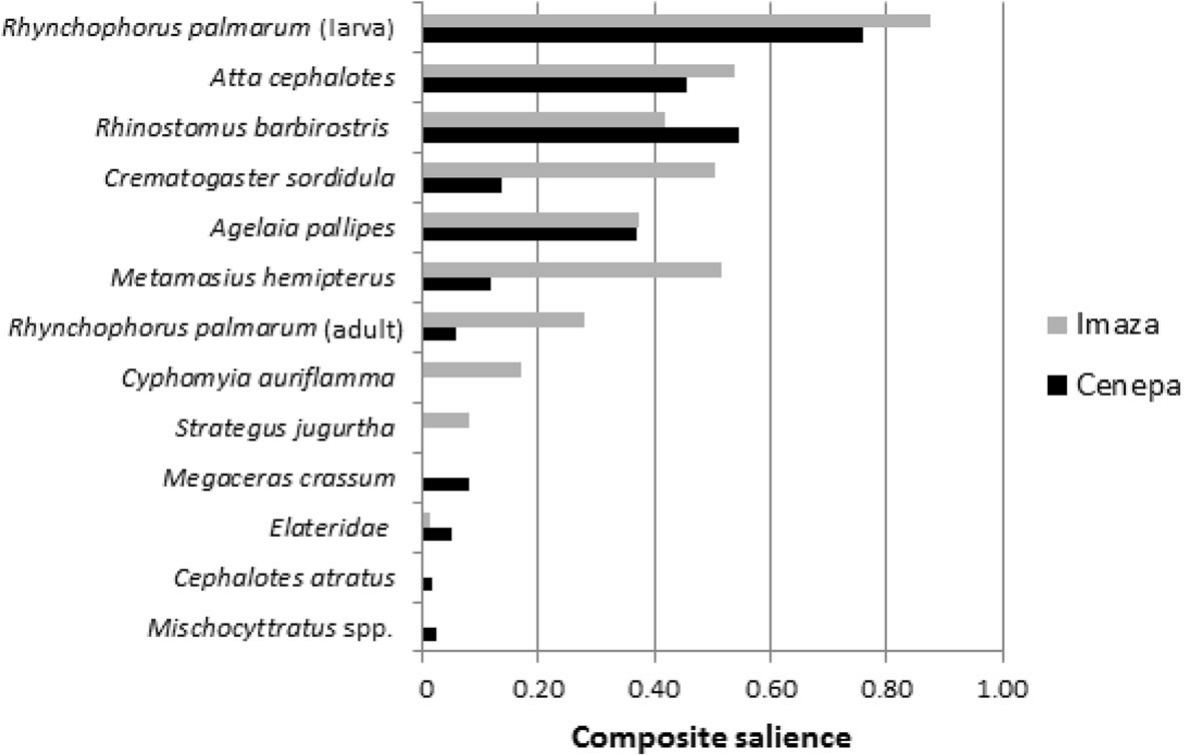
Insects’ cultural importance in the Cenepa and Imaza districts

By comparing the districts, the most significant differences in the insects’ cultural importance were the cases of *Metamasius hemipterus* Linnaeus, *C*. *sordidula*, and *R*. *palmarum* (adult), which all obtained higher salience indices in the Imaza district (Fig. 2). Considering species consumed solely in a particular district, Imaza has two unique species (*C*. *auriflamma* and *S*. *jugurtha*), whereas three species are specific to Cenepa (*C*. *atratus*, *Mischocyttarus* sp., and *M*. *crassum*).

Regarding the number of freelisted insects, in Imaza, people listed 6.6 insects on average, while in Cenepa, the average was lower (4.2). However, the total number of listed insects was 10 in Imaza and 11 in Cenepa.

In Imaza, remarkably, 100% of the respondents mentioned *R*. *palmarum* as the most consumed insect. In Cenepa, this species was indicated as the most consumed by 86% of the respondents, and in contrast with Imaza, *M*. *hemipterus* larvae are also a major insect food in the district. In Cenepa, *A*. *pallipes* pupae too play an important role in the diet of 27% of the respondents, but this is true for only 3% of the respondents in Imaza. A reverse proportion was found sometimes with regard to *M*. *hemipterus* larvae, which are preferred by 23% of respondents in Imaza, compared to 2% of the respondents in Cenepa. Of all of the insects, nine were indicated as the most consumed in Imaza, compared with 11 in Cenepa.

### Collection patterns and associated knowledge

In *Awajún* culture, men are the most important insect collectors (57% of households), followed by women (17.5%), and then by both men and women (15%). Traditional knowledge about insect collection is usually transmitted vertically through the parents. Fathers teach insect collection in 64% of the households, mothers in 18%, both parents in 15%, and grandparents in 3%. The collection patterns depend on particular species, although most are collected manually with the help of the tools such as axes and machetes used to cut the insects’ host plants. The most common techniques of collecting insects include tree felling (46%), handpicking (42%), and burning (12%). Coleoptera larvae such as *R*. *palmarum*, *R*. *barbirostris*, and *M*. *hemipterus* are mostly collected from trunks of intentionally felled or naturally fallen palms of *Mauritia flexuosa* L. (male individuals) and *Attalea phalerata* Mart. ex Spreng. This practice is considered to be semi-cultivation because 1 or 2 months after felling, people return to harvest the larvae by hand collection. The *M*. *hemipterus* larvae are collected commonly also from the palm *Astrocaryum chambira* Burret, while larvae of the unidentified species from Elateridae family are collected exclusively from the *M*. *flexuosa*. *M*. *crassum*, and *S*. *jugurtha* in their adult stages (beetle) are collected from their host plant *Gynerium sagittatum* (Aubl.) P.Beauv. (Poaceae). Hymenoptera are captured, depending on the species. Terrestrial ants are collected in large numbers using torches set up to attract the insects when the reproductive castes (males and queens) emerge, starting their nuptial flights. They are attracted by the light, trapped, and stored in a sack. Other ants that build their nests in trees such as *C*. *sordidula* and *C*. *atratus* are harvested through feeling the host trees’ trunks and branches; then, they are collected manually and deposited in a small container. The method used to collect the wasps comprises burning down the nest to eliminate adults and harvest the pupae. Diptera larvae are harvested by hand from the same species of palm trees from which Coleoptera larvae are collected.

### Seasonal availability

Coleoptera and Diptera larvae are available from January to December (Table 1). Hymenoptera availability is more seasonal. While tree ants are available all year round, terrestrial ants are collected solely from September to October. Wasp pupae and beetle adults are collected exclusively during the dry season between June and September. Importantly, the most culturally significant insects, *R*. *palmarum* larvae, are available almost year-round, but *A*. *cephalotes*, the second most important insect, is available only for a 2-month period.

### Insect preparation and consumption

**Fig. 3:**
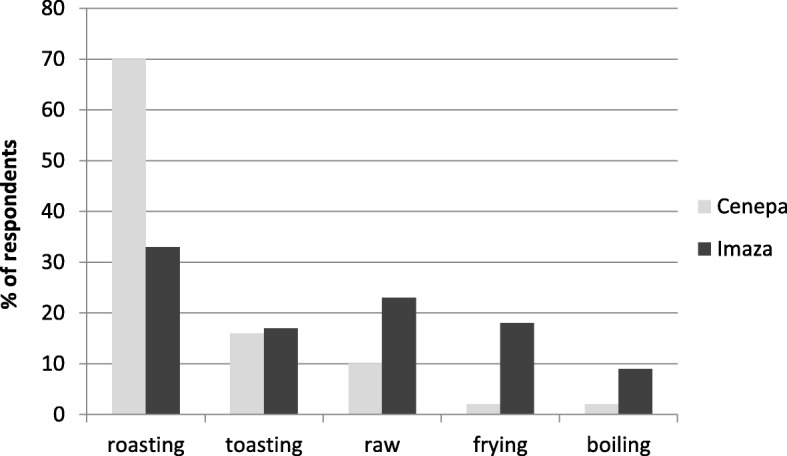
Culinary insect preparation methods in Cenepa and Imaza districts

Edible insects in *Awajún* communities are prepared as a food in different ways. The most common method of preparation is roasting (67%), the method when hot coal and ash are spread uniformly and then insects are placed over a grid and roasted. In the toasting method (10%), the wood fire is used but in a low heat and the insects are placed in a pan until a crispy texture is obtained. Frying (5%) is the main method used to prepare palm weevil larvae, which are washed and then fried in a pan. The larvae exude their own fat during the frying process, so there is no need for addition of cooking oil. Boiling (2%) is also used for the preparation of palm weevil larvae, when prepared together with local vegetables and aromatic herbs, and the resultant broth is consumed as a soup. Nevertheless, a certain proportion (16%) of the insects investigated is also consumed raw. Significant differences were found between the two districts studied. While, in Cenepa, insects were overwhelmingly roasted and, to a lesser extent, toasted or consumed raw, in Imaza, all recorded insect preparation practices were nearly equally applied (Fig. 3).

The beetles are consumed according to their developmental stages. The larvae can be prepared roasted on a wood stick, as part of a broth or as a salad mixed with palm hearts (*iju*). Adults are usually consumed toasted and grilled. The traditional dish *patarashca* represents another form of preparation. In this case, *R*. *palmarum* or *R*. *barbirostris* larvae are wrapped in the *bijao* [*Calathea lutea* (Aubl.) E. Mey. ex Schult., Marantaceae] leaf and cooked on coal. Hymenoptera consumption habits differ according to species. Ants are mainly consumed toasted and fried, whereas wasps are consumed roasted.

## Discussion

According to Jongema [6], over 700 insect species have been reported as a food resource from the Neotropics to date with predominant orders being Coleoptera, Hymenoptera, Lepidoptera, and Isoptera, which remains far from the estimates of Paoletti et al. [29], who stated for Hymenoptera 600 species consumed in South America and Coleoptera thousands of species consumed in the Amazon. Peru is one of the Latin American countries where insect consumption is an important component of the indigenous people’s traditional food [30]. Unfortunately, no comprehensive scientific study documenting food-use patterns of insects in the Peruvian context has been published to date. A unique study of Creed-Kanashiro et al. [16] in different *Awajún* communities in Peru found a large diversity of fish and other animals hunted but only three insect species (Coleopteran palm grub, hymenopteran *Brachygastra* sp., and one formicid species).

Our survey identified 12 insect species, 5 of which reported for the first time as a food resource (3 Hymenoptera, 1 Coleoptera, and 1 Diptera). The higher insect number cited by the respondents having more children might indicate that insects play a more important role for larger families. The association with income could be influenced by the insects’ economic value because 40% of our respondents were both consuming and selling insects. When comparing knowledge and insect consumption between the districts of Cenepa and Imaza, we may assume that in Imaza, the traditional entomological knowledge is distributed more equally. Therefore, even though Cenepa is richer in locally edible insects, individual knowledge is lower. Although, in Imaza, people on average knew more edible insects, their food preference was narrower and focused predominantly on *R. palmarum*. Meanwhile, in Cenepa, more insect species represent important food sources. This result is somewhat surprising considering the higher number of edible insects freelisted in Imaza, yet it may show the potential gap between knowledge and practical use. The salience index (Table 1) which is commonly used to demonstrate plant cultural significance in ethnobotanical studies was adopted to demonstrate the importance of individual insect species in this ethnoentomological study. According Quinlan [26], the strength of the Salience Index (S) is that it considers both frequency of mentioning as well as position in the list (prominence, familiarity, and representativeness). This index has been applied in several previous studies of traditional entomological knowledge in, e.g., Nepal by Björnsen [31] and Lima et al. in Brazil [32].

Looking at the documented species’ nutritional content, we were only able to find nutritional characteristics for two species (*R. palmarum*, *A. cephalotes*). Considering the average nutritional values of insects in different taxonomical orders (Table 3), the cultural salience of the insect orders in the *Awajún* food system (Table 2) tends to increase with the contents of total fat and energy. It might indicate the people’s preference for collecting and consuming energy-dense insects. Our literature review showed that in Latin America, the larva of *R. palmarum* contain on average of 6.6 g of protein/100 g of fresh weight, while the ant *A. cephalotes* contain on average 50.4 g of protein/100 g of dry weight (Table 3).

**Table 3:** Energy value and macronutrient composition of different insect taxa

Insect taxa	Energy [Kcal]*	Protein [g]*	Total fat [g]*	CH^⁑^ [g]*
Order				
Coleoptera	283-653 [49]	50.41 (23–66) [50]	25.57 (14–36) [50]	2.81 [50]
Hymenoptera	380–561 [49]	47.81 (13–77) [50]	21.42 (8–55) [50]	3.65 (2–7) [50]
Diptera	217–499 [49]	59.39 [50]	12.61 [50]	12.04 [50]
Species				
*R. palmarum* (larva)	188 (125–273) [12, 18, 51]	6.57 (1.4–13.06) [12, 18, 19, 51, 52]	13.10 (6.31–21.96) [12, 18, 19, 51, 52]	7.69 [12, 18, 19, 51]
*Atta cephalotes*	454 (390–580) [40, 49, 53]	50.4 (43–60.11) [40, 49, 53]	28.4 (25.8–31) [40, 49]	24 [49]

*The values for *Rhynchophorus palmarum* are on average per 100 g of fresh weight, the other values could not be found or converted to fresh weight, and therefore are given on average per 100 g of dry weight. In parentheses is the range of variability found in the literature

^⁑^*CH* carbohydrates

Regarding nutrient composition, protein from insects is highly digestible, and insects contain a number of nutritionally valuable amino acids including considerable amounts of phenylalanine and tyrosine. Moreover, some insects contain significant levels of important amino acids threonine, and lysine, which are deficient in certain plant proteins and thus plant-based diets [33]. Fat from insects contains a proportion of beneficial poly-unsaturated fatty acids. According to Chakravorty et al. [34] in the ant *Oecophylla smaragdina* Fabricius, the mono-unsaturated fatty acids fraction (51.55%) dominates the lipids, followed by saturated fatty acids (40.26%) and poly-unsaturated fatty acids (8.19%). Insects are also rich in several micronutrients such as copper, iron, magnesium, manganese, phosphorous, and zinc. Nevertheless, they are notoriously poor suppliers of the essential amino acid methionine, vitamin A, vitamin C, niacin, and thiamine [35]. However, the nutrient composition of insects is highly dependent on its feed.

Our study indicated that Coleoptera include the most popular edible insect species consumed across Amazonian ethnic groups [36]. Coleoptera are the most species-rich order of insects with 360–400 edible species known worldwide, described and accepted [37]. Beside the well-known species *R*. *palmarum* and relatively common *R*. *barbirostris* [38], our study documented two lesser-known species of palm weevils *M*. *hemipterus* and one unidentified elaterid species.

The second most culturally salient insect species in the present study was the ant *A*. *cephalotes*, which the *Awajún* consumed in its adult stages. *Atta* ants as a food resource have previously been recorded for the *Tukanoans* in southeastern Colombia [39] and among many other Amazonian tribes [29]. In addition to palm weevils, *A*. *cephalotes* is known to be a food resource rich in energy and crude protein. Palm weevils and *Atta* spp. are also most commonly consumed in other parts of the Amazon because they form large and highly predictable aggregations [40].

The Coleopteran larvae of *R*. *palmarum*, *R*. *barbirostris*, and *M*. *hemipterus* are usually obtained through a semi-cultivation practice based on intentional felling or naturally fallen palms of *M*. *flexuosa* (almost exclusively male individuals of this dioecious palm), *A*. *phalerata* and less frequently from *A*. *chambira*, *Oenocarpus bataua* Mart., and *Bactris gasipaes* Kunth. The use of both *R*. *palmarum* and *R*. *barbirostris* species has been observed in *Jotï* communities [38] and the *Yanomami* tribe in Venezuela [41]. According to Choo et al. [38], the *Jotï* cultivate *R*. *palmarum* and *R*. *barbirostris* larvae in the same trunks of the *Oenocarpus bacaba* Mart. palm. The Yanomani collect the same larvae from two preferred palm species: *O*. *bataua* or *M*. *flexuosa* [41]. A recent study of the *Guarani*, in northeastern Argentina, reported a semi-cultivation of larvae of three species from Dryophthoridae (*M. hemipterus*, *R. palmarum* and *R. barbirostris*) in the stems of the palm *Syagrus romanzoffiana* [5]. However, one of the most important host plant for the palm weevils is *M*. *flexuosa*, which has recently been put under pressure because of the overharvesting of its edible fruits, widely commercialized in the Peruvian Amazon [42]. To lower the potential overexploitation risk of *M*. *flexuosa*, in Venezuela, Cerda et al. [36] tested three palm-based substrates (*Maximiliana maripa* Mart., *Jessenia bataua* Mart., *M*. *flexuosa*) to cultivate *R. palmarum* and found that the most nutrient-dense larvae were obtained from the *M. flexuosa*, yet the content of micronutrients was higher in larvae grown on the *M. maripa* and *J. bataua*.

In accordance with the *Jivi* tribe in Venezuela [36], Coleoptera in their adult developmental stages (beetles) are also consumed among the *Awajún* in Peru, namely, *M*. *crassum* and *S*. *jugurtha* hosted by the invasive grass *G*. *sagittatum*.

Regarding the collection patterns, handpicking was the most common method of gathering insects reported in the present study. This method is common also among the *Yanomamo* Indians who collect arboreal termites of the genus *Nasutitermes* [41] and the *Enawenê-Nawê* Indians of the State of Mato Grosso for the ant *A*. *cephalotes* [7]. The *Awajún* people in our study reported a combination of handpicking with torchlight to attract the soil insects, i.e., *Atta*. The method of burning down the nest reported in our study is practiced similarly for all wasps in Brazil [43] and Venezuela [41], since they are very ferocious.

In the Amazon, the edible insect’s availability is highly seasonal. For example, Choo et al. [38] state that the *Jotï* Indians of the Venezuelan Amazon report that the optimal period of palm weevil growth starts at the end of the rainy season and ends at the beginning of the dry season (September–January). Delgado et al. [18] conclude that the best time to obtain palm-associated insects in the Peruvian Amazon is the dry season from June to August. According to our study, seasonality in general is an important factor influencing food availability and food intake in the study area. For example, most wasps were consumed during the dry season, which is the period of ovipositional and wasp larval growth; hence, this is the best time for egg, larval, and pupal collection [44]. According to the *Awajún’s* traditional entomological knowledge, the best time to harvest is during the full moon time, but the *Popolocas* Indians in Mexico collect wasp nests only when the moon is between its last quarter and waning gibbous, a period when nests are full of larvae and honey [45]. The *Awajún* consume *A*. *cephalotes* exclusively during their mating period when individuals are fertile and at the beginning of the rainy season (September–October). Araujo and Becerra [41] reported that in the Venezuelan Amazon, the *Yanomami* and *Yekuana* Indians also consume ants in the same season.

Regarding insect preparation methods, the *Awajún* applied various modes. These include roasting, toasting, frying, and boiling, with roasting being the most popular method. Earlier studies reported modified modes of preparation of edible insects in the Amazon, e.g., the *Tukuna* and *Tapirapé* Amazon Indians consume ant and wasp larvae roasted and mixed with cassava flour, which traditionally accompanies all foods with an animal origin consumed there [7]. It should be mentioned that the consumption of wild animals could be risky for human health, because it may transmit some diseases (zoonoses) [46]. A recent systematic review deals with zoonotic agents of meat and other by-products of wild species used as food such as reptiles, rodents, ungulates, and primates, among others, in tropical and subtropical regions [47]. In our study, no health problems related to the consumption of insects were observed, but further research on safety and hygienic handling of edible Amazonian insects is strongly recommended [48].

## Conclusions

The *Awajún* communities have developed a rational insect resource management, applying sustainable collection and consumption patterns with occasional trading in the case of abundance. Energy-dense insects, which form large and predictable aggregations, tend to be used more commonly. Beetles (particularly grubs) are the most culturally important edible insects, followed by ants, wasps, and flies. In the more accessible and developed Imaza district, the *Awajún* tend to eat almost exclusively *R*. *palmarum*, while in the more isolated and preserved Cenepa district, the preferences are linked with more species. In the future, certain species of beetles such as *R. palmarum*, *R*. *barbirostris*, and *M*. *hemipterus* appear to be particularly appropriate for scaling up a fledgling production as they are already being semi-cultivated locally. This survey discovered five species that are newly recorded as edible (*A*. *pallipes*, *C*. *atratus*, *C*. *sordidula*, *C*. *auriflamma*, and *S*. *jugurtha*). The missing nutritional characteristics should be complemented by a laboratory analysis and awareness regarding the insects’ importance for the communities’ nutrition should be raised to tap the potential of this traditional food resource.

## Abbreviations

CH: Carbohydrates
IGA: Internal Grant Agency of the Faculty of Tropical AgriSciences, Czech University of Life Sciences Prague
LP: Lukas Pawera
ODECOFROC: *Organización de Desarrollo de las Comunidades Fronterizas del Cenepa*
PPVP: Pablo Pedro Villegas Panduro
RCR: Rubén Casas Reátegui
S: Salience Index
UNIA: *Universidad Nacional Intercultural de la Amazonia*
ZP: Zbynek Polesny

## References

[1] FAO (2015). The state of food insecurity in the world 2015 - meeting the 2015 international hunger targets: taking stock of uneven progress.

[2] INEI-Instituto Nacional de Estadística e Informática. Encuesta Demográfica y de Salud Familiar 2016. Lima: Nacional y Regional. Chapter II. 2017. p. 30–1. https://www.inei.gob.pe/media/MenuRecursivo/publicaciones_digitales/Est/Lib1433/index.html. Accessed 15 Nov 2017.

[3] Meyer-Rochow VB (1975). Can insects help to ease the problem of world food shortage?. Search.

[4] Posey DA (1987). Ethnoentomological survey of Brazilian Indians. Entomol Gener.

[5] Araujo JJ, Keller HA, Hilgert NI. Management of pindo palm (*Syagrus romanzoffiana*, Arecaceae) in rearing of Coleoptera edible larvae by the Guarani of Northeastern Argentina. Ethnobiol Conserv. 2018; 10.15451/ec2018017.01118.

[6] Jongema Y. List of edible insects of the world. Wageningen: Wageningen University. 2017. https://www.wur.nl/en/Research-Results/Chair-groups/Plant-Sciences/Laboratory-of-Entomology/Edible-insects/Worldwide-species-list.htm. Accessed 28 Nov 2017.

[7] Costa Neto EM, Ramos-Elorduy J (2006). Edible insects of Brazil ethnicity, diversity and relevance as human food. Bol Soc Entomol Aragon.

[8] Rivas AX, Pazos SC, Castillo SKC, Pachón H (2010). Alimentos Autóctonos de las Comunidades Indígenas y Afrodescendientes de Colombia. Arch Latinoam Nutr.

[9] Berlin EA, Markell EK. An assessment of the nutritional and health status of an Aguaruna Jivaro community, Amazonas, Peru. Ecol Food Nutr. 1977; 10.1080/03670244.1977.9990483.

[10] Roca AF. Las Palmeras en el Conocimiento Tradicional del Grupo Indígena Amazónico Aguaruna-Huambisa. Rev Peru Biol. 2008; 10.15381/rpb.v15i3.3773.

[11] Samaniego GD. Las Misiones del Alto Marañón: Evangelización Aguaruna-Humbisas Desde la Espiritualidad Ignaciana. Master’s Thesis, Facultad de Teología, Universidad Pontificia Comillas, Madrid, Spain. 2016. http://hdl.handle.net/11531/10143. Accessed 22 Nov 2017.

[12] Creed-Kanashiro HM, Carrasco M, Abad M, Tuesta I, Kuhnlein HV, Erasmus B, Spigelski D, Burlingame B (2013). Promotion of traditional foods to improve the nutrition and health of the Awajún of the Cenepa River in Peru. Indigenous peoples’ food systems: the many dimensions of culture, diversity and environment for nutrition and health.

[13] Roche ML, Creed-Kanashiro HM, Tuesta I, Kuhnlein HV. Traditional food system provides dietary quality for the Awajún in the Peruvian Amazon. Ecol Food Nutr. 2007; 10.1080/03670240701486651. 10.1017/S1368980007000560 17610756

[14] Lepofsky D. The past, present, and future of traditional resource and environmental management. J Ethnobiol. 2009; 10.2993/0278-0771-29.2.161.

[15] RAISG. Deforestación en la Amazonía (1970-2013). Red Amazónica de Información Socioambiental Georeferenciada. 2015. https://www.amazoniasocioambiental.org/es/publicacion/deforestacion-en-la-amazonia-1970-2013-atlas/. Accessed 2 May 2017.

[16] Creed-Kanashiro HM, Roche M, Tuesta CI, Kuhnlein HV, Kuhnlein HV, Erasmus B, Spigelski D, Burlingame B (2009). Traditional food system of an Awajun community in Peru. Indigenous peoples’ food systems: the many dimensions of culture, diversity and environment for nutrition and health.

[17] Huamán-Espino L, Valladares C (2006). Estado Nutricional y Características del Consumo Alimentario de la Población Aguaruna,. Amazonas, Perú. 2004. Rev Peru Med Exp Salud Publica.

[18] Delgado C, Couturier G, Mathews P, Mejia K (2008). Producción y Comercialización de la Larva de Rhynchophorus palmarum (Coleoptera: Dryophthoridae) en la Amazonía Peruana. Bol Soc Entomol Aragon.

[19] Vargas GE, Espinoza G, Ruiz C, Rojas R (2013). Valor Nutricional de la Larva de Rhynchophorus palmarum L.: Comida Tradicional en la Amazonía Peruana. Rev Soc Quím Perú.

[20] Wolverton S, Nolan JM, Ahmed W. Ethnobiology, political ecology, and conservation. J Ethnobiol. 2014; 10.2993/0278-0771-34.2.125.

[21] Jernigan KA. The importance of chemosensory clues in Aguaruna tree classification and identification. J Ethnobiol Ethnomed. 2008; 10.1186/1746-4269-4-12. 10.1186/1746-4269-4-12 PMC2412849 18454871

[22] Dávila PJ. Perú: Gobiernos Locales y Pueblos Indígenas. Lima: Tarea Gráfica Educativa. 2005. https://centroderecursos.cultura.pe/. Accessed 5 Oct 2017.

[23] Costa Neto EM, Santos Fita D, Vargas CM (2009). Manual de Etnozoología. Una Guía Teórico-Práctica para Investigar la Interconexión del Ser Humano con los Animales.

[24] Bernard HR (2002). Research methods in anthropology: qualitative and quantitative approaches.

[25] ICZN-International Commission on Zoological Nomenclature. 2017. http://www.iczn.org/iczn/index.jsp. Accessed 10 Oct 2017.

[26] Quinlan M. Considerations for collecting Freelists in the field: examples from Ethnobotany. Field Methods. 2005; 10.1177/1525822X05277460.

[27] Smith JJ. Using ANTHOPAC 3.5 and a Spreadsheet to Compute a Free-List Salience Index. Field Methods. 1993; 10.1177/1525822X9300500301.

[28] FAO/INFOODS (2012). Guidelines for Converting Units, Denominators and Expressions, Version 1.0.

[29] Paoletti MG, Buscardo E, Dufour DL. Edible invertebrates among amazonian indians: a critical review of disappearing knowledge. Environ Dev Sustain. 2000; 10.1023/A:1011461907591.

[30] Costa Neto EM. Edible insects in Latin America: old challenges, new opportunities. J Insects Food Feed. 2016; 10.3920/JIFF2016.x001.

[31] Björnsen AG (2003). Insects – a mistake in God’s creation? Tharu farmers’ perception and knowledge of insects: a case study of Gobardiha Village development committee, dang-Deukhuri, Nepal. Agr Hum Values.

[32] Lima DCO, Ramos MA, da Silva HCH, Alves AGC. Rapid assessment of insect fauna based on local knowledge: comparing ecological and ethnobiological methods. J Ethnobiol Ethnomed. 2016; 10.1186/s13002-016-0085-z. 10.1186/s13002-016-0085-z PMC4774136 26932264

[33] Kouřimská L, Adámková A. Nutritional and sensory quality of edible insects. NFS J. 2016; 10.1016/j.nfs.2016.07.001.

[34] Chakravorty J, Ghosh S, Megu K, Jung C, Meyer Rochow VB. Nutritional and anti-nutritional composition of Oecophylla smaragdina (Hymenoptera: Formicidae) and Odontotermes sp. (Isoptera: Termitidae): Two preferred edible insects of Arunachal Pradesh, India. JAP Entomol. 2016; 10.1016/j.aspen.2016.07.001.

[35] Rumpold BA, Schlüter OK. Nutritional composition and safety aspects of edible insects. Mol Nutr Food Res. 2013; 10.1002/mnfr.201200735. 10.1002/mnfr.201200735 23471778

[36] Cerda H, Martinez R, Briceno N, Pizzoferrato L, Manzi P, Tommaseo Ponzetta M, Marin O, Paoletti MG. Palm worm (*Rhynchophorus palmarum*): traditional food in Amazonas, Venezuela. Nutritional composition, small scale production and tourist palatability. Ecol Food Nutr. 2001; 10.1080/03670244.2001.9991635.

[37] Chapman AD (2009). Numbers of living species in Australia and the world. Report for the Australian Biological Resources Study.

[38] Choo J, Zent EL, Simpson BB. The importance of traditional ecological knowledge for palm-weevil cultivation in the Venezuelan Amazon. J Ethnobiol. 2009; 10.2993/0278-0771-29.1.113.

[39] DeFoliart GR. An overview of the role of edible insects in preserving biodiversity. Ecol Food Nutr. 1997; 10.1080/03670244.1997.9991510.

[40] Dufour DL. Insects as food: a case study from Northern Amazon. Am Anthropol. 1987; 10.1525/aa.1987.89.2.02a00070.

[41] Araujo Y, Beserra P (2007). Diversity of invertebrates consumed by the Yanomami and Yekuana communities from the Alto Orinoco, Venezuela. Interciencia.

[42] Gilmore MP, Endress BA, Horn CM. The socio-cultural importance of *Mauritia flexuosa* palm swamps (aguajales) and implications for multi-use management in two Maijuna communities of the Peruvian Amazon. J Ethnobiol Ethnomed. 2013; 10.1186/1746-4269-9-29. 10.1186/1746-4269-9-29 PMC3733440 23607601

[43] Costa Neto EM (2004). La Etnoentomología de las Avispas (Himenóptera, Vespoidea) en el Poblado de Pedra Branca, Estado de Bahía, Nordeste de Brasil. Boletín Sociedad Entomológica Aragonesa..

[44] Rodríguez-Jiménez A, Sarmiento CE. Altitudinal distribution and body resource allocation in a High Mountain social wasp (Hymenoptera: Vespidae). Neotrop Entomol. 2008; 10.1590/S1519-566X2008000100001. 10.1590/s1519-566x2008000100001 18368243

[45] Acuña AM, Caso L, Aliphat MM, Vergara CH. Edible insects as part of the traditional food system of the Popoloca town of Los Reyes Metzontla, Mexico. J Ethnobiol. 2011; 10.2993/0278-0771-31.1.150.

[46] Still J (2003). Use of animal products in traditional Chinese medicine: environmental impact and health hazards. Complem Therap Medic.

[47] Van Vliet N, Moreno J, Gómez J, Zhou W, Fa JM, Golden C, Alves RRN, Nasi R. Bushmeat and human health: assessing the evidence in tropical and sub-tropical forests. Ethnobiol Conserv. 2017; 10.15451/ec2017-04-6.3-1-45.

[48] Jansson A, Berggren A (2015). Insects as food – something for the future? A report from Future Agriculture.

[49] Ramos-Elorduy J, Pino JM, Prado EE, Perez MA, Otero JL, de Guevara OL. Nutritional value of edible insects from the state of Oaxaca, Mexico. J Food Compos Anal. 1997; 10.1006/jfca.1997.0530.

[50] Xiaoming C, Ying F, Hong Z, and Zhiyong Ch. Review of the Nutritive Value of Edible Insects. In: Durst PB, Johnson DV, Leslie RN and Shono K, editors. Forest Insects as Food: Human Bite Back. Proceedings of a Workshop on Asia-Pacific Resources and their Potential for Development. Bangkok: FAO; 2010. http://www.fao.org/docrep/012/i1380e/i1380e00.pdf. Accessed 2 Dec 2017.

[51] Cerda H, Martinez R, Briceño N, Pizzoferrato L, Hermoso D, Paoletti MG (1999). Rearing, nutritional composition, and sensorial analysis of the Rhynchophorus palmarum (Coleoptera: Curculionidae) palm weevil as a food eaten by the Amazonian Indians. Ecotropicos.

[52] Sanchez P, Jaffe K, Hevia P (1997). Consumo de Insectos: Alternativa Proteíca del Neotrópico. Bol Entomol Venez.

[53] Ramos-Elorduy J, Moreno JMP (2001). El Consumo de Insectos Entre los Lacandones de la Comunidad Bethel y su Valor Nutritivo. Etnobiología.

